# Using marketing strategies to improve recruitment and retention in clinical trials: a scoping review

**DOI:** 10.1186/s13063-026-09576-9

**Published:** 2026-03-03

**Authors:** Antony Ndungu, Kirsty Sprange, Elizabeth R. Nixon, Eleanor J. Mitchell

**Affiliations:** 1 Nottingham Clinical Trials Unit, School of Medicine, University Park Campus, University of Nottingham, Nottingham, NG7 2RD UK; 2 Nottingham University Business School, Jubilee Campus, University of Nottingham, Nottingham, NG8 1BB UK

**Keywords:** Trials, Recruitment, Retention, Marketing

## Abstract

**Background:**

Participant recruitment and retention into randomised trials (RCTs) is key to the success of any trial yet remains a major challenge. Recruiting and retaining trial participants is akin to an organisation trying to engage with new audiences. Marketing is one way in which organisations engage with audiences as a strategic approach to create, communicate, deliver and exchange offerings of value to consumers. Clinical trials could benefit from strategically implementing a broad array of principles and practices from marketing theory. It could enable trialists to better understand how to improve the value of the trial experience for the various stakeholders involved i.e. participants, recruiting sites, clinicians, and policy makers.

The aim of this scoping review was to investigate the landscape of marketing use in clinical trials literature to inform future and best practice. This work is part of a wider programme of work aimed at developing a marketing toolkit to support people designing and running clinical trials.

**Methods:**

Medline (OVID), Cochrane Library, CINAHL (EBSCO), Web of Science, SSRN, Clinicaltrials.gov and Google Scholar databases were searched for relevant articles. Eligible articles were screened by independent reviewers. An extraction table was created and used in Covidence to extract and manage the data. The scoping review was conducted and is reported using PRISMA-ScR.

**Results:**

A total of 61 articles were eligible and included in the review. Most (75%, *n* = 46) of the articles reported on marketing used in the context of trial recruitment, while 25% (*n* = 15) focused on both recruitment and retention. No articles focused solely on retention. Varied marketing activities and approaches were described. Promotional aspects of marketing accounted for the vast majority of all reported activities. Social media and online advertising were the most reported promotional activities. The use of multiple promotional activities in a trial was reported to be beneficial in improving recruitment.

**Conclusion:**

Trial teams are already utilising different marketing techniques with the aim of improving recruitment and retention. However, there is a selective concentration on promotional techniques especially advertising, which limits the potential of marketing to improve recruitment and retention of participants.

**Supplementary Information:**

The online version contains supplementary material available at 10.1186/s13063-026-09576-9.

## Background

Randomised controlled trials (RCTs) are integral to evidence-based medicine and should be designed and conducted to high standards to generate quality evidence. Participant recruitment and retention are pivotal to the success of every RCT, yet they remain some of the most reported challenges [1, 2]. Failing to recruit enough participants results in a low sample size and ultimately low statistical power [3]. Poor retention, especially loss of participants before the collection of key outcomes, lowers the probability of detecting the effect from an intervention resulting in unreliable RCT findings [4]. Unmet recruitment targets and poor retention therefore result in underpowered and unreliable RCTs, increased trial costs and contribute to research waste [3, 5, 6].

A review of publicly funded UK RCTs conducted between 1997 and 2020 found that only 63% (245/388) of RCTs achieved their sample size, and 32% (128/388) of RCTs required a time extension in their recruitment period. Additionally, 30% (118/388) of RCTs required a sample size revision [5]. Retention also poses a significant challenge for trial teams, as the same review reported an 88% median retention rate, estimating at least a 10% loss of recruited participants [5].

One approach that could improve participant recruitment and retention in RCTs could be via greater understanding of the marketing discipline. Francis et al. argued that clinical trials are akin to a business seeking to engage customers, while trial teams strive to engage clinicians and patients to participate in clinical trials [7]. Marketing has a debatable reputation, often associated with selling and economic gain. However, fundamentally, marketing as a discipline is more than selling—it seeks to understand audiences or consumers’ perspectives and what they value. The central objective in marketing is matching the needs and expectations of an audience with offers of value specifically suited to meet those needs and expectations [8, 9]. Trial teams have multiple target audiences: patients, participants, health professionals, recruiting sites and other stakeholders such as regulatory and commissioning bodies. Each group may have different expectations of the trial experience. Using marketing could help prioritise understanding of the different target audience’s perspective with an aim to provide a better experience for them. This could enhance the value and appeal of RCTs to improve recruitment and retention [1, 7, 9]. Although implementing principles and practices from the marketing discipline could be helpful in recruitment and retention of participants in RCTs [1, 7, 9], evidence on how marketing principles are applied in clinical trials is limited and further evidence is needed to inform future and best practice.

We aimed to systematically map the evidence contextualising the use of marketing in recruitment and retention in RCTs, as well as identify the gaps in knowledge and how these gaps could be potentially addressed by conducting a scoping review. The principal question for this scoping review was “How is marketing applied and utilised in participant recruitment and retention in randomised clinical trials?”.

## Methods

### Study design

The scoping review was conducted in line with the JBI methodology for scoping reviews and reported using Preferred Reporting Items for Systematic reviews and Meta-Analyses extension for Scoping Reviews (PRISMA-ScR) [10, 11]. The protocol was published on Open Science Framework (OSF) prior to data extraction [12].

### Inclusion criteria

The Participant, Concept, Context (PCC) framework was used to develop the inclusion criteria.

Participants/population: there were no specific inclusion/exclusion criteria relating to participants/population. Evidence from all reported populations was considered important.

Concept: the use of marketing in recruitment and retention of RCT participants. Articles needed to explicitly examine marketing activities and/or principles used to enhance recruitment and retention in randomised clinical trials. Articles were included whether they discussed conceptual frameworks, implemented strategies, or reviewed effectiveness of applied marketing activities.

Context: articles reporting RCTs from any clinical speciality in any setting across the world.

### Exclusion

Articles were excluded if they were not available in English either originally or through translation. There were no restrictions on publication dates.

### Search strategies

The search strategy was developed in consultation with an expert medical librarian at the University of Nottingham. Using a combination of Medical Subject Headings (MeSH) and controlled vocabulary, journal articles and other published material were identified from international databases. The research question was interdisciplinary, so literature was searched from both medical and social science databases including Medline (OVID), Cochrane Library, CINAHL (EBSCO), Web of Science, ASSIA **(**ProQuest), Clinicaltrials.gov, and Social Science Research Network (SSRN) from database inception to March 2024. A search was also conducted on Google Scholar for grey literature.

The MeSH terms and query filters were applied and adjusted according to the requirements of each electronic database. A detailed search strategy was published with the protocol [12]. Sample search done on Medline (Ovid) is attached in supplementary material 1.

### Evidence selection

Following the literature search, duplicates and titles that did not meet the inclusion criteria were removed. The remaining citations were imported into EndNote version 20™, a reference management tool for the next stage of review.

Initial screening included an assessment of the titles prior to retrieval of the full text articles. Full text articles were retrieved and their abstracts screened by independent reviewers. AN was the first reviewer of all articles. Articles were then equally distributed between other authors (ERN, KS, EJM) for a second review. For an article to proceed from one stage to the next stage, two reviewers needed to agree on its eligibility. Finally, full text articles were reviewed against the inclusion/exclusion criteria. Where two reviewers could not agree, all reviewers convened to discuss any uncertainties or disagreements and gain consensus on the inclusion or exclusion of an article. Articles that met all the criteria were imported into Covidence™, a systematic review management tool for extraction.

### Data extraction

Eligible articles were extracted for data including the year of publication, the article’s country of origin, the article methodology and study design, population described in the article, the marketing activities being reported and the reported merits and disadvantages of the marketing activities.

We identified marketing as what authors labelled as “marketing”. This included any strategic activities or approaches reported in the articles that trial teams used to communicate, promote, raise awareness and build relationships with patients, participants or communities aimed at improving recruitment and retention in clinical trials (for example social media advertising, posters and flyers). The data extraction process was iterative and discussed by reviewers. We identified marketing activities in context as described in Table 1 below.

**Table 1:** Marketing activities identified a priori

Type of marketing	Types of activities involved/reported
Newspaper/magazine marketing	Advertisements or communications in newspapers and magazines
TV/Radio marketing	Airing advertisements and communications on television and radio
Out of home marketing	Advertising to participants while they are outside of their homes, including billboards, advertisements on street furniture such as free-standing ad panels and bus shelters
Email marketing	Mass or personalised/targeted emails sent to mailing lists/registers
Print marketing	Use of posters, flyers
Social media marketing	Advertisements or communications on Facebook, X, YouTube, Snapchat, Instagram and other social media platforms
Online marketing	The use of search-related ads, display ads on a website
Social marketing	Interventions for behaviour change that improves individual wellbeing and that of society such as safety campaigns
Mobile marketing	The use of short message services (SMS) and mobile applications
Direct mail	Mailouts and communications sent via post
Influencer marketing/endorsement	Trial teams partnering with individuals/professionals with credibility and an audience such as healthcare workers, and care givers
Other reported marketing	These included in-person, community recruitment and public outreach events

To ensure consistency and accuracy of data extraction, a piloting phase was conducted on 16% (*n* = 10) of the articles. Each author reviewed a subset of the articles. From all identified articles, AN initially extracted ten articles, ERN and EJM each reviewed three of these articles, while KS reviewed four. We resolved any identified issues and clarifications through further discussions by all the reviewers. The data extraction tool was modified twice during the pilot process before agreement was reached on the final template.

All data extraction was then completed by AN. After the extraction process, 10% (*n* = 6) of all articles extracted that had not been included in the pilot phase were selected at random (every 10th article from a list in alphabetical order) and independently reviewed by KS. This process successfully verified the extracted data was valid and consistent.

### Data analysis

We analysed and summarised data quantitatively and in a narrative synthesis.

#### Quantitative analysis

Quantitative data were imported into Statistical Packages for Social Sciences (SPSS) version 29.0 for analysis and summarised using frequency counts and percentages. Data were presented in chart and tabular formats.

#### Narrative synthesis

**Fig. 1:**
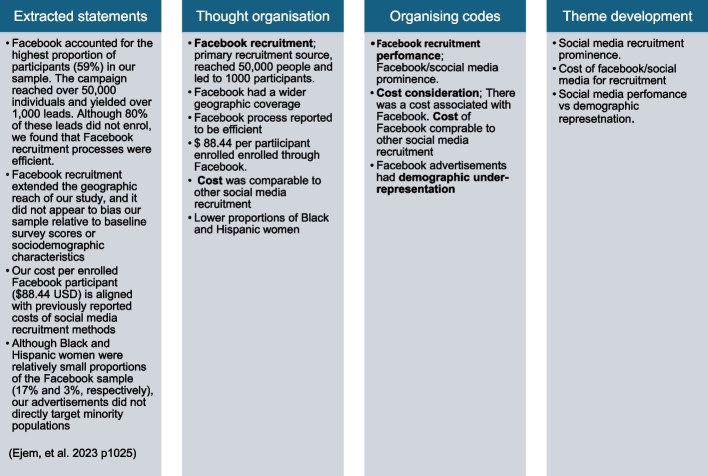
Sample of the synthesis process

Textual data on the use of marketing activities in recruitment and retention was synthesised using descriptive content analysis [13]. Textual data that were synthesised included the reported marketing activity, its reported benefits and the reported limitations in the recruitment and retention of trial participants. AN read and became familiarised with the textual data before proceeding to code the data line by line. The coding process was iterative and identified points of interest in relation to the review’s objectives. Figure 1 provides an example of the synthesis process.

Articles used in the data extraction pilot were used to create and refine the coding framework. After all articles were coded, similar codes and categories were merged and grouped into broader themes.

## Results

**Fig. 2:**
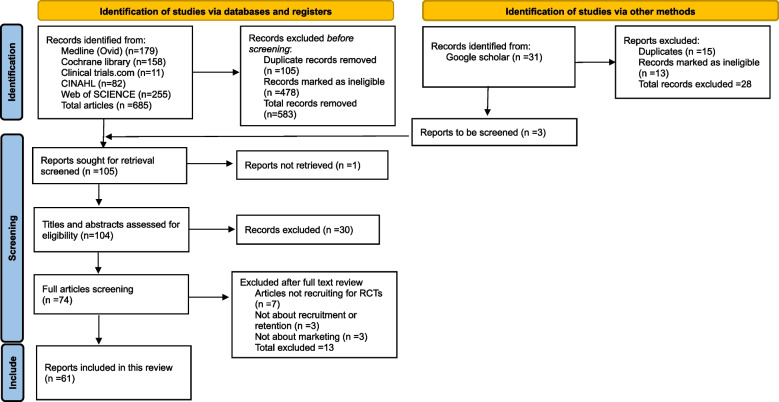
PRISMA diagram

Sixty-one articles (supplementary material 2) met the inclusion criteria and were included (Fig. 2).

**Fig. 3:**
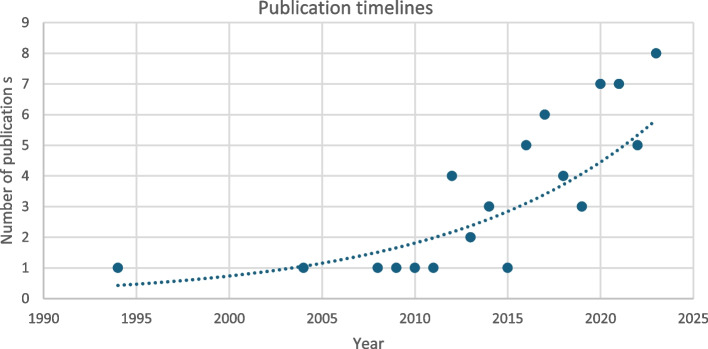
Timeline of publications 1990–2023

The number of publications reporting marketing in clinical trials has gradually risen over time with a notable increase over the last decade (Fig. 3).

Most articles originated from developed countries, with North America, Europe and Oceania accounting for 67%, 20% and 12% respectively.

There was a wide variation in the methodologies of articles included. Of the 61 articles included, 66% (*n* = 40) were articles linked to or nested in larger RCTs, including retrospective observations of recruitment and retention outcomes. Seventy-five per cent (*n* = 46) of the articles reported on marketing activities used in the context of clinical trial recruitment, while 25% (*n *= 15) focused on both recruitment and retention aspects. No articles focused solely on retention. Almost all articles (*n* = 55, 90%) described marketing aspects that had either been implemented or were being evaluated, and only 10% (*n* = 6) focused on conceptual/theoretical frameworks of marketing use in clinical trials. Almost all identified activities were promotional aspects of marketing such as advertising, except for social marketing which uses all elements of the marketing mix to influence voluntary behaviour change [14].

The type of marketing activities reported varied, with most of the articles reporting more than one activity being undertaken (Table 2). Social media and online advertising were the most reported promotional activities while out-of-home methods such as billboards were least reported.

**Table 2:** Reported marketing activities

Marketing activities	Published articles *n* = 61 (%)
Social media advertising	39 (66)
Online advertising (search-related ads, ads on a website)	28 (48)
Print marketing (posters, flyers)	21 (36)
Newspaper/magazine advertising	16 (27)
In person and community recruitment, public outreach events	15 (25)
Social marketing	14 (24)
TV/Radio Ads	12 (20)
Email marketing (emails sent to mailing list/register)	9 (15)
Direct mail (post)	9 (15)
Mobile marketing	3 (5)
Influencer marketing	3 (5)
Out-of-home advertising	1 (2)

### Reported benefits and limitations of marketing use in RCTs

1. Social media and online marketing

Forty-five articles referred to either social media or online advertising when detailing the type of marketing they employed. Social media platforms varied across the articles with platforms such as Facebook, Instagram and Reddit, being reported. Other online platforms used for recruitment in specific populations included classified advertising platforms such as Craigslist and online dating websites such as Grindr. Facebook was the most common social media platform cited. Social media activities were depicted as beneficial and versatile recruitment tools with a wide reach and an ability to target specified populations of interest from the general population.

Facebook accounted for the highest proportion of participants (59%) in our sample. The campaign reached over 50,000 individuals and yielded over 1,000 leads. Ejem et. Al 2023 [15].

Facebook advertisements can be more targeted than many of the traditional approaches because it is possible to define specific demographic and geographic characteristics, and information can be directed to those who search terms on the Web that align with the interests specified by the researcher [16].

Social media and online advertising use in trials were not without caveats. It was reported that when used for participant recruitment, they are likely to yield a non-representative sample. For example, they were reported as potentially more appealing to a younger demographic who are more likely to have an online presence, and they were also reported to be less attractive to ethnic minority groups.

Facebook recruited participants, and their parents were more likely to be younger, and more likely to report their child’s ethnicity as white and as such be less diverse…. Buckler et al. 2023 [17].

The proportions of eligible contacts who identified as a racial or ethnic minority were lower for those recruited through online advertising as compared to other recruitment efforts, while the proportions of eligible contacts who were under the age of 18 was higher [18].

Another reported caveat for social media and online advertisements was privacy and confidentiality concerns. Articles [19–22] raised concerns about social media and online platforms posing a privacy risk by misusing personal data and data mining for targeted advertisement campaigns and security risks posed by data leaks.

2. Multi-method approach

Generally, articles reported varied use of promotional activities in recruitment and retention based on a study’s context and its participants. Using multiple promotional activities was suggested to be more effective. Articles that utilised a combination of recruitment strategies, for example supplementing online methods such as social media with traditional offline methods such as flyers, posters and in-person strategies, reported it to be more effective in reaching a more diverse group than using a single method.

Therefore, utilizing the combination of traditional recruitment approaches and social media–based approaches is ideal to avoid this selection bias [16].

…we believe that a combination of modern marketing strategies and more traditional recruitment strategies through existing research networks offers a way of improving primary care trial recruitment. Colwell et. [23, 24].

Social media and clinical site recruitment are complementary strategies that appear to draw in families with different profiles… Buckler et. Al 2023 [17].

3. Partnerships and collaboration

Although authors did not explicitly discuss partnerships and collaborations as marketing, they emerged as an important theme. Partnerships and collaborations were described as ways of promoting trials to target audiences and linking various stakeholders leading to improved participant recruitment into trials. Articles reported the benefits of partnerships between research teams and community organisations, healthcare providers and patient groups. Dumas et al. [25] andAlHeresh et al. [26] reported positive outcomes during recruitment by partnering with doctors’ practices to reach their target audience.

Etkin et al. [27] reported that they were able to meet and exceed their recruitment goal of 30% minority ethnic groups in their sample through collaborations with a multicultural outreach programme. Applequist et al. [28] worked with a patient advocacy group and asked their representatives to review all recruitment messages. Their feedback was incorporated into the final recruitment materials which the advocacy group then shared on their platforms.

…the PAG’s (patient advocacy group) willingness to share the RDCRN’s recruitment message on its own social media platform with its followers meant that an already established, engaged, and targeted audience was provided in advance, which likely influenced the chances of the recruitment being effective. [28].

4. Cost of marketing

Cost was reported as an influential factor in which marketing techniques were employed. Cost varied significantly across different promotional options in participant recruitment. More traditional forms of media such as broadcast (TV and radio) were reported as being more expensive with a relatively lower yield [29]. Social media appears to have mixed return on investment with some articles [30–32] reporting it to be cost-effective while others [33] reporting the contrary.

To yield good value, authors commonly stated that promotional activities needed optimal implementation often requiring time in planning and a certain level of expertise which was also factored in as a cost.

Our research team had little experience with Facebook advertisements before starting the study. The advertisements might have been more impactful or cost-effective if those formulating them had more experience developing, testing, and monitoring social media platforms and related statistics [16].

Facebook/Instagramadverts were more expensive to create, with additional expertise required to support the successful launch and maintenance of the trial ad campaigns. Haydock et. Al, 2023 [34].

## Discussion

We explored published evidence on how marketing is applied in clinical trials to inform future use and best practice. The findings indicate that the use of marketing in participant recruitment and retention into RCTs is not a novel concept. Indeed, trial teams are already considering different marketing activities and utilising them in varying modalities in the process of recruitment and/or retention of participants in clinical trials. Our review suggests the use of marketing in clinical trials has grown and that trial teams are embracing technological advancements, specifically social media and online channels, especially over the last decade.

However, we found a greater focus on recruitment over retention strategies. This is despite both recruitment and retention being identified as the highest priorities for RCT methodology research in the UK [35]. Retention has also been recognised by the European Medicines Agency (EMA) and Food and Drug Administration (FDA) as a priority for RCT methodology [36, 37]. Our review suggests that more work is therefore required to understand how marketing can be used to improve retention specifically. Despite the extensive focus on recruitment, uncertainty remains in matching recruitment activities to target populations and the resources and expertise to maximise recruitment activities.

We found that although trial teams are already employing marketing techniques in the trial recruitment process, this is almost exclusively limited to the promotional aspect of marketing. In marketing practice, activities such as advertising, online presence and direct mail are known as ‘marketing communications’ and sit within the promotion element of the 4Ps marketing mix (Product, Price, Place and Promotion) [38]. Even though marketing and advertising are often mentioned and referenced together, it is salient to recognise that advertising is just one tool within marketing and that the discipline offers a much wider range of ideas and tools that can be used to improve recruitment and retention, beyond advertising, for example, value exchange may be influential in participant recruitment [9], and Customer Relationship Management (CRM) may be helpful in maintaining relationships with participants, hence improving retention. Marketing and advertising are distinct and serve different roles and purposes [39, 40].

Marketing as a discipline considers offering value to target audiences as a paramount component [41]. Clinical trials can be viewed as an important service with clinical and social value. They generate high-quality evidence that is directly relevant to clinicians, patients and health policy makers [42, 43]. Beyond the broader contributions, participants can also benefit directly through gaining access to treatments not available outside of trial settings, close monitoring, follow-up care and health education from clinical and research teams [44]. Marketing also considers consumer satisfaction as a key factor and an important predictor for loyalty and intention to re-consume a service or a product [41]. Considering marketing beyond promotion could help trial teams deliberate on improving the trial experience for the various stakeholders. This could help mitigate avoidable issues that deter potential participants and sites from participating in trials, improve retention and lead to improved working relationships with sites.

We found that social media and online advertisements were the most frequently reported marketing activities, respectively. Justifiably, social media and online channels have the capacity to do both mass marketing and direct marketing. This yields the ability to reach a greater audience and have more engagement with potential participants. Additionally, these channels can be interactive and increase interest in a trial potentially leading to participation. The unidirectional communication of most traditional and offline promotional methods means they are limited in this respect [45]. Most social media platforms require users to sign up using a personal profile. This gives the platforms an extensive database of user data profiles. This makes social media highly effective for targeting a specific audience. For example, Facebook can allow a trial team to set up a campaign targeting a specific age group of a single gender in a specific geographic location. Additionally, some platforms have machine learning models that enable predictive targeting based on a user’s activities which further increase their targeting potential [46].

Our results suggest that social media can lead to recruitment of a less generalisable population. Teenagers and young adults tend to have a larger online presence, and social media platforms may prioritise their needs over older people [47]. However, social media use for older audiences should not be overlooked as there is evidence to suggest that Facebook may be an effective recruitment tool for an older audience while Instagram and TikTok may be better for a younger audience [48]. Ethnic and racial groups’ use of social media is varied, with literature suggesting an overrepresentation of ethnically white people and females in health research recruitment [49]. People from lower social economic communities are likely to have lower digital literacy and potentially also be underrepresented in social media and online recruitment [50]. All these factors considered, social media and online recruitment require prudent and ethical usage.

Our results reflect existing literature that multiple yet integrated marketing activities improve the likelihood of improving participant recruitment and retention [51]. Various factors such as age, ethnicity and other contextual factors are influential in the trial recruitment process. Marketing activities vary in their ability to appeal and recruit different populations; some approaches will appeal better to certain populations than others. Therefore, it is important to acknowledge the variations within the population of interest while implementing marketing campaigns [52]. Trial teams need to clearly and prudently define, identify and divide their target groups from the general population and even within the trial itself. This is known as audience segmentation in marketing [53]. Segmentation helps in planning strategic, efficient and effective marketing campaigns around defined groups [52]. Accurate segmentation may be particularly useful for understanding the needs and values of underserved groups and reaching them.

Our results noted the importance of partnerships and collaborations with clinicians, patient advocacy groups and community groups. Similarly, Dunleavy et al. in their systematic review exploring social marketing framework in RCTs reported working with partners and partner education as an enabler in overcoming gatekeepers in palliative RCTs [54]. Trial teams could benefit from collaboration with their target audiences and entities who are stakeholders or gatekeepers to the populations of interest from the outset before the trial design stage. Partnerships and co-production efforts take many forms and are characterised differently, including “patient and public involvement and engagement” (PPIE). The people or partners involved in the production and delivery of a service have a strong influence on how consumers perceive a service and its quality. This is denoted as people or personnel in the 7Ps (Product, price, place, promotion, personnel, process and physical evidence) of the services marketing mix [24, 38]. Interactions with tailored communication, advice and exchange of ideas between trial teams and identified partners can effectively demonstrate the optimum value of a trial to target audiences [24]. PPIE activities are indispensable and many clinical trial funders require strong PPIE in trial design and conduct to be described and demonstrated at the time of funding application [55]. Early and adept consultations, partnership and co-production are not only important for improving recruitment efforts, but can also have a positive effect on reducing power imbalances between researchers and communities, build trust and reciprocity, subsequently increasing the overall impact of health research including clinical trials [56].

Justifiably, cost was an important factor in the choice and use of marketing activities in our results. Trials can be expensive; hence, optimising costs and reducing waste is essential. In the RCT lifecycle, participant recruitment can be one of the most expensive stages, estimated to consume up to a third of a trial’s budget and being one of the largest drivers of the increased trial costs [57, 58]. We found that marketing costs were reported to vary considerably across the range of activities. The variance in cost of recruitment per participant could be explained by myriad factors and circumstances, such as inter- and intra-platform differences, paid versus unpaid media, time and the human resources required for a marketing activity. Consequently, this makes the cost of marketing a trial highly contextual based on the population of interest and specific nuances of the trial. Cost per participant is only one dimension of value; other contextual factors should be considered. For instance, in-person and community outreach recruitment was depicted as being resource-intensive, often requiring additional staff as well as being time-consuming, leading to an overall increased cost per participant [26, 59]. However, in-person engagement could provide opportunities to build relationships, collaborations and trust and could be crucial to reaching target groups especially underserved communities leading to improved recruitment and retention. This value may justify the cost of in-person activities. For trial teams, the challenge lies in selecting the right mix of tools and channels for marketing within an RCT.

The strengths of this scoping review are a well-developed and thorough search strategy, independent reviewers and methodological validation through piloting phases and secondary independent checks at various stages. Additionally, the team of reviewers comprised of a multi-disciplinary team with clinical trial and marketing expertise.

Our scoping review also had some limitations. We developed a broad search strategy across various databases to identify articles. However, it is possible we could have missed some relevant articles. Though we used strategies such as collaborator expertise and experience to define and identify what can be termed as a marketing activity in the context of clinical trials, it is possible that others might recommend the inclusion or exclusion of different activities.

## Conclusion

This scoping review aimed to assess how marketing practices and principles are currently applied in clinical trials to inform future use. Based on the results, we conclude that various promotional activities from marketing are used in attempts to improve recruitment and retention efforts. However, other techniques from marketing such as gathering participant feedback are not conventionally used but could be very beneficial. Social media and online advertisements are the most reported methods due to their wide reach, ability to target, and costs.

Complementary marketing activities should be used together due to their varied ability to appeal to different populations. When considering marketing activities and developing marketing materials for RCTs, it is important for trial teams to define and identify their target audiences and partner with stakeholders representing these audiences to increase their reach and success in recruitment efforts. Cost is an important factor in the consideration of marketing activities. In an effort to lower trial costs, trial teams are keen on low-cost marketing techniques with a high yield.

Trial recruitment and retention ultimately rely on willingness to participate. However, marketing makes a difference in engagement, building relationships, increasing awareness, driving recruitment and providing a satisfactory clinical trial experience for participants and other stakeholders.

## Supplementary Information

Additional file 1. Search strategy for Medline (Ovid).

Additional file 2. All included articles.

Additional file 3. PRISMA-ScR-Checklist.

## Data Availability

Summary data are presented in the manuscript and supplementary material. Other data are available from the corresponding author on reasonable request.

## Abbreviations

JBI: Joanna Briggs Institute
OSF: Open Science Framework
PCC: Participant, Concept, Context
PPIE: Patient and public involvement and engagement
PRISMA-ScR: Preferred Reporting Items for Systematic reviews and Meta-Analyses extension for Scoping Reviews
RCTs: Randomised controlled trials
SPSS: Statistical Packages for Social Sciences
UK: United Kingdom
