# Supplementary figures and images for: Using marketing strategies to improve recruitment and retention in clinical trials: a scoping review
Source: Trials. 2026 Mar 3;27:269. doi: 10.1186/s13063-026-09576-9 (PMC13045075; doi:10.1186/s13063-026-09576-9)

**Supplementary material 1: Search strategy for Medline (Ovid)**


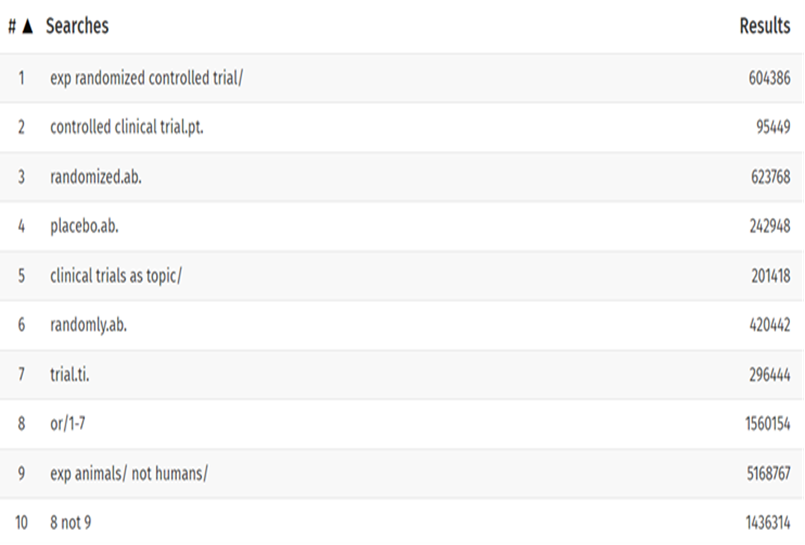


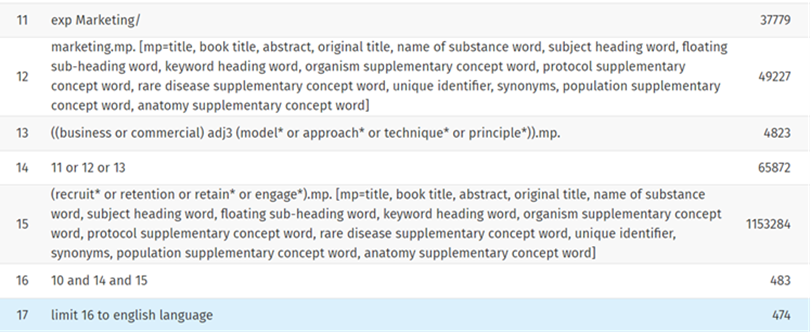

Supplement: Supplementary file 1 — Additional file 1. Search strategy for Medline (Ovid). [file 13063_2026_9576_MOESM1_ESM.docx]
