# Supplementary material for: Using marketing strategies to improve recruitment and retention in clinical trials: a scoping review
Source: Trials. 2026 Mar 3;27:269. doi: 10.1186/s13063-026-09576-9 (PMC13045075; doi:10.1186/s13063-026-09576-9)
Supplement: Supplementary file 2 — Additional file 2. All included articles. [file 13063_2026_9576_MOESM2_ESM.docx]

**Supplementary material 2: All included articles**

| **Authors and study year.** | **Title** |
| --- | --- |
| Adam, L. M., Manca, D. P., & Bell, R. C. (2016) | Can Facebook be used for research? Experiences using Facebook to recruit pregnant women for a randomized controlled trial |
| Akers, L., & Gordon, J. S. (2018) | Using Facebook for large-scale online randomized clinical trial recruitment: effective advertising strategies |
| AlHeresh, R., Vaughan, M. W., Brenner, I. H., & Keysor, J. (2021) | Recruitment cost and outcomes for an arthritis work disability prevention randomized clinical trial: The Work It study |
| Applequist, J., Burroughs, C., Ramirez, A., Merkel, P. A., Rothenberg, M. E., Trapnell, B., Desnick, R. J., Sahin, M., & Krischer, J. P. (2020) | A novel approach to conducting clinical trials in the community setting: utilizing patient-driven platforms and social media to drive web-based patient recruitment |
| Applequist, J., Burroughs, C., Merkel, P. A., Rothenberg, M., Trapnell, B., Desnick, R., Sahin, M., & Krischer, J. (2023) | Direct-to-Consumer Recruitment Methods via Traditional and social media to Aid in Research Accrual for Clinical Trials for Rare Diseases: Comparative Analysis Study |
| Baker, A., Mitchell, E. J., & Thomas, K. S. (2022) | A practical guide to implementing a successful social media recruitment strategy: lessons from the Eczema Monitoring Online trial |
| Bracken, K., Hague, W., Keech, A., Conway, A., Handelsman, D. J., Grossmann, M., Jesudason, D., Stuckey, B., Yeap, B. B., Inder, W., Allan, C., McLachlan, R., Robledo, K. P., & Wittert, G. (2019) | Recruitment of men to a multi-centre diabetes prevention trial: an evaluation of traditional and online promotional strategies |
| Buckingham, L., Becher, J., Voytek, C. D., Fiore, D., Dunbar, D., Davis-Vogel, A., Metzger, D. S., & Frank, I. (2017) | Going social: Success in online recruitment of men who have sex with men for prevention HIV vaccine research |
| Buckler, E. J., Gonzalez, O.-J., Ball, G. D. C., Hamilton, J., Ho, J., Morrison, K. M., & Masse, L. C. (2023) | Recruiting families using social media versus pediatric obesity clinics: a secondary analysis of the Aim2Be RCT |
| Byrd-Bredbenner, C., Delaney, C., Martin-Biggers, J., Koenings, M., & Quick, V. (2017) | The marketing plan and outcome indicators for recruiting and retaining parents in the HomeStyles randomized controlled trial |
| Campbell, A. N. C., Fisher, D. S., Picciano, J. F., Orlando, M. J., Stephens, R. S., & Roffman, R. A. (2004) | Marketing effectiveness in reaching the nontreatment-seeking marijuana smoker |
| Cho, E., Gilmer, M. J., Friedman, D. L., Hendricks-Ferguson, V. L., Hinds, P. S., & Akard, T. F. (2021) | Facebook Recruitment for Children with Advanced Cancer and Their Parents: Lessons from a Web-based Pediatric Palliative Intervention Study |
| Colwell, B., Mathers, N., Ng, C. J., & Bradley, A. (2012) | Improving recruitment to primary care trials: some lessons from the use of modern marketing techniques |
| Dumas, A.-A., Lemieux, S., Lapointe, A., Provencher, V., Robitaille, J., & Desroches, S. (2020) | Recruitment and retention of mothers of preschoolers and school-aged children in a social media-delivered healthy eating intervention: lessons learned from a randomized controlled trial |
| Dunleavy, L., Walshe, C., Oriani, A., & Preston, N. (2018) | Using the 'Social Marketing Mix Framework' to explore recruitment barriers and facilitators in palliative care randomised controlled trials? A narrative synthesis review |
| Dunsiger, S. I., Bock, B. C., Horowitz, S., Lantini, R., Jennings, E., & Thind, H. (2022) | Advertising Effectiveness for Recruitment and Retention in a Trial of Yoga for Smoking Cessation |
| Ejem, D. B., Wechsler, S., Gallups, S., Khalidi, S., Coffee-Dunning, J., Montgomery, A. P., Stevens, C. J., Keene, K., Rocque, G. B., Chamberlin, M., Hegel, M. T., Azuero, A., Pisu, M., Ellis, D., Ingram, S. A., Lawhon, V. M., Gilbert, T., Morrissette, K., Morency, J., . . . Lyons, K. D. (2023) | Enhancing Efficiency and Reach Using Facebook to Recruit Breast Cancer Survivors for a Telephone-Based Supportive Care Randomized Trial During the COVID-19 Pandemic |
| Etkin, C. D., Farran, C. J., Barnes, L. L., & Shah, R. C. (2012) | Recruitment and enrollment of caregivers for a lifestyle physical activity clinical trial |
| Faro, J. M., Nagawa, C. S., Orvek, E. A., Smith, B. M., Blok, A. C., Houston, T. K., Kamberi, A., Allison, J. J., Person, S. D., & Sadasivam, R. S. (2021) | Comparing recruitment strategies for a digital smoking cessation intervention: technology-assisted peer recruitment, social media, ResearchMatch, and smokefree.gov |
| Frampton, G. K., Shepherd, J., Pickett, K., Griffiths, G., & Wyatt, J. C. (2020) | Digital tools for the recruitment and retention of participants in randomised controlled trials: a systematic map |
| Frandsen, M., Walters, J., & Ferguson, S. G. (2014) | Exploring the viability of using online social media advertising as a recruitment method for smoking cessation clinical trials |
| Galli, L., Knight, R., Robertson, S., Hoile, E., Oladapo, O., Francis, D., & Free, C. (2014) | Using marketing theory to inform strategies for recruitment: a recruitment optimisation model and the txt2stop experience |
| Gimble, J., Kline, L., Makris, N., Muenz, L., & Friedman, L. (1994) | Effects of new brochures on blood donor recruitment and retention |
| Gioia, C. J., Sobell, L. C., Sobell, M. B., & Agrawal, S. (2016) | Craigslist versus print newspaper advertising for recruiting research participants for alcohol studies: Cost and participant characteristics |
| Grafeo, M., Key, K. (2023) | Crossing the Divide: How nontraditional approaches can transform clinical trial recruitment. |
| Graham, A. L., Cha, S., Cobb, N. K., Fang, Y., Niaura, R. S., & Mushro, A. (2013) | Impact of Seasonality on Recruitment, Retention, Adherence, and Outcomes in a Web-Based Smoking Cessation Intervention: Randomized Controlled Trial |
| Gupta, A., Calfas, K. J., Marshall, S. J., Robinson, T. N., Rock, C. L., Huang, J. S., Epstein-Corbin, M., Servetas, C., Donohue, M. C., Norman, G. J., & et al. (2015) | Clinical trial management of participant recruitment, enrollment, engagement, and retention in the SMART study using a Marketing and Information Technology (MARKIT) model |
| Guthrie, K. A., Caan, B., Diem, S., Ensrud, K. E., Greaves, S. R., Larson, J. C., Newton, K. M., Reed, S. D., & LaCroix, A. Z. (2019) | Facebook advertising for recruitment of midlife women with bothersome vaginal symptoms: A pilot study |
| Haydock, R., Hepburn, T., Ross, J., & Wilson, J. (2023) | Online advertising - going viral: online advertising on the deva bacterial vaginosis randomised controlled trial |
| Hayes, T., & Sharma, M. (2021) | Applying the integrated marketing communication approach to recruit and retain African American women |
| Hoffmann, S. H., Folker, A. P., Buskbjerg, M., Folker, M. P., Jezek, A. H., Svarta, D. L., Sølvhøj, I. N., & Thygesen, L. (2022) | Potential of Online Recruitment Among 15-25-Year Olds: Feasibility Randomized Controlled Trial |
| Iannotti, L. L., Gallegos‐Riofrío, C. A., Waters, W. F., Carrasco, A. M., Salvador, J. M., Lutter, C. K., & Stewart, C. P. (2018) | The Lulun Project's social marketing strategy in a trial to introduce eggs during complementary feeding in Ecuador |
| Jones, R. B., Goldsmith, L., Williams, C. J., & Kamel Boulos, M. N. (2012) | Accuracy of geographically targeted internet advertisements on Google AdWords for recruitment in a randomized trial |
| Jones, R., Lacroix, L., & Porcher, E. (2017) | Facebook Advertising to Recruit Young, Urban Women into an HIV Prevention Clinical Trial |
| Juraschek, S. P., Plante, T. B., Charleston, J., Miller, E. R., Yeh, H.-C., Appel, L. J., Jerome, G. J., Gayles, D., Durkin, N., KarenWhite, Dalcin, A., & Hermosilla, M. (2018) | Use of online recruitment strategies in a randomized trial of cancer survivors |
| Kira, A., Glover, M., Walker, N., & Bauld, L. (2016) | Recruiting Pregnant Indigenous Women Who Smoke into a High Contact Incentivized Cessation Trial: A Feasibility Study |
| Kutok, E. R., Doria, N., Dunsiger, S., Patena, J. V., Nugent, N. R., Riese, A., Rosen, R. K., & Ranney, M. L. (2021) | Feasibility and Cost of Using Instagram to Recruit Adolescents to a Remote Intervention |
| Ladd, D. L., & Wright, J. C. (2023) | Promoting rural residents' participation in clinical trials: clinical trials basics programming and training for rural public librarians |
| Lang, S. R., Day, K., Gallaher, E., Jebeile, H., Collins, C. E., Baur, L. A., & Truby, H. (2023) | Participant recruitment for paediatric research using social media: A practical 'how-to' guide for researchers |
| LeBlanc, T. W., Lodato, J. E., Currow, D. C., & Abernethy, A. P. (2013) | Overcoming recruitment challenges in palliative care clinical trials |
| Lee, H., Hübscher, M., Moseley, G. L., Kamper, S. J., Traeger, A. C., Skinner, I. W., Williams, C. M., & McAuley, J. H. (2017) | An embedded randomised controlled trial of a Teaser Campaign to optimise recruitment in primary care |
| Lopez, E. N., Simmons, V. N., Quinn, G. P., Meade, C. D., Chirikos, T. N., Brandon, T. H., Lopez, E. N., Simmons, V. N., Quinn, G. P., Meade, C. D., Chirikos, T. N., & Brandon, T. H. (2008) | Clinical trials and tribulations: lessons learned from recruiting pregnant ex-smokers for relapse prevention |
| McAnulty, J. D. (2009) | Bringing Patient Recruitment into Our Digital World |
| McDonald, A. M., Treweek, S., Shakur, H., Free, C., Knight, R., Speed, C., & Campbell, M. K. (2011) | Using a business model approach and marketing techniques for recruitment to clinical trials |
| McKenzie, P. L., Siegel, D. H., Cullen, D., & Castelo-Soccio, L. (2021) | Strategies to enhance pediatric clinical trial participation: A systematic review with narrative synthesis |
| Miller, H. N., Plante, T. B., Gleason, K. T., Charleston, J., Mitchell, C. M., Miller, E. R., 3rd, Appel, L. J., & Juraschek, S. P. (2021) | A/B design testing of a clinical trial recruitment website: A pilot study to enhance the enrollment of older adults |
| Mitchell, E. J., Sprange, K., Treweek, S., & Nixon, E. (2022) | Value and engagement: what can clinical trials learn from techniques used in not-for-profit marketing? |
| Nash, E. L., Gilroy, D., Srikusalanukul, W., Abhayaratna, W. P., Stanton, T., Mitchell, G., Stowasser, M., & Sharman, J. E. (2017) | Facebook advertising for participant recruitment into a blood pressure clinical trial |
| Nouvini, R., Parker, P. A., Malling, C. D., Godwin, K., & Costas-Muniz, R. (2022) | Interventions to increase racial and ethnic minority accrual into cancer clinical trials: A systematic review |
| Prescott, T. L., Phillips Ii, G., DuBois, L. Z., Bull, S. S., Mustanski, B., & Ybarra, M. L. (2016) | Reaching Adolescent Gay, Bisexual, and Queer Men Online: Development and Refinement of a National Recruitment Strategy |
| Reuter, K., Ukpolo, F., Ward, E., Wilson, M. L., & Angyan, P. (2016) | Trial Promoter: a web-based tool for boosting the promotion of clinical research through social media |
| Rudkowski, J. L., Pond, G. R., Tremblay, A., Johnston, M., Goss, G., Nicholas, G., Martel, S., Bhatia, R., Liu, G., Schmidt, H., Tammemagi, M. C., Atkar-Khattra, S., Tsao, M.-S., Lam, S., & Goffin, J. R. (2020) | Trial marketing in the Pan-Canadian Early Detection of Lung Cancer Study |
| Salvy, S.-J., Carandang, K., Vigen, C. L. P., Concha-Chavez, A., Sequeira, P. A., Blanchard, J., Diaz, J., Raymond, J., & Pyatak, E. A. (2020) | Effectiveness of social media (Facebook), targeted mailing, and in-person solicitation for the recruitment of young adult in a diabetes self-management clinical trial |
| Staffileno, B. A., Zschunke, J., Weber, M., Gross, L. E., Fogg, L., & Tangney, C. C. (2017) | The Feasibility of Using Facebook, Craigslist, and Other Online Strategies to Recruit Young African American Women for a Web-Based Healthy Lifestyle Behavior Change Intervention |
| Tate, D. F., LaRose, J. G., Griffin, L. P., Erickson, K. E., Robichaud, E. F., Perdue, L., Espeland, M. A., & Wing, R. R. (2014) | Recruitment of young adults into a randomized controlled trial of weight gain prevention: message development, methods, and cost |
| Whiteley, J. A., Faro, J. M., Mavredes, M., Hayman, L. L., & Napolitano, M. A. (2021) | Application of social marketing to recruitment for a digital weight management intervention for young adults |
| Withall, J., Jago, R., & Fox, K. R. (2012) | The effect a of community-based social marketing campaign on recruitment and retention of low-income groups into physical activity programmes-a controlled before-and-after study |
| Witzel, T. C., Gabriel, M. M., McCabe, L., Weatherburn, P., Gafos, M., Speakman, A., Pebody, R., Burns, F. M., Bonell, C., Lampe, F. C., Dunn, D. T., Ward, D., Harbottle, J., Phillips, A. N., McCormack, S., & Rodger, A. J. (2019) | Pilot phase of an internet-based RCT of HIVST targeting MSM and transgender people in England and Wales: advertising strategies and acceptability of the intervention |
| Wray, T. B., Chan, P. A., Klausner, J. D., Ward, L. M., Liu, A. Y., Carr, D. J., Ocean, E. M., Phelan, C., & Liu, T. (2023) | Using web analytics data to identify platforms and content that best engage high-priority HIV populations in online and social media marketing advertisements |
| Yu, P., & Waller, K. (2010) | M matters: What's social marketing and media got to do with it? |
| Zlotorzynska, M., Bauermeister, J. A., Golinkoff, J. M., Lin, W., Sanchez, T. H., & Hightow-Weidman, L. (2021) | Online recruitment of youth for mHealth studies |
